# Supplementary material for: Circulating Levels of SMPDL3B Define Metabolic Endophenotypes and Subclinical Kidney Alterations in Myalgic Encephalomyelitis
Source: Int J Mol Sci. 2025 Sep 12;26(18):8882. doi: 10.3390/ijms26188882 (PMC12470165; doi:10.3390/ijms26188882)
Supplement: Supplementary file 1 [file ijms-26-08882-s001.zip › ijms-3802051-SI.pdf]

Circulating Levels of SMPDL3B Define Metabolic Endophenotypes and Subclinical Kidney Alterations in Myalgic Encephalomyelitis

Bita Rostami-Afshari [1,2,3,4], Wesam Elremaly [2,3,4], Neil R. McGregor [5,6,7], Katherine Jin Kai Huang [5,7], Christopher W. Armstrong [5,7,*], Anita Franco [2,3,4], Christian Godbout [4], Mohamed Elbakry [2,3,4,8], Rim Abdelli [9] and Alain Moreau [1,2,3,4,10,*]

1 Department of Biochemistry and Molecular Medicine, Faculty of Medicine, Université de Montréal, Montreal, QC H3T 1J4, Canada
2 Viscogliosi Laboratory in Molecular Genetics of Musculoskeletal Diseases, Azrieli Research Center, CHU Sainte-Justine, Montreal, QC H3T 1C5, Canada
3 Open Medicine Foundation ME/CFS Collaborative Center, CHU Sainte-Justine/Université de Montréal, Montreal, QC H3T 1C5, Canada
4 ICanCME Research Network, Azrieli Research Center, CHU Sainte-Justine, Montreal, QC H3T 1C5, Canada
5 Department of Biochemistry and Molecular Biology, Bio21 Molecular Science and Biochemistry Institute, 30 Flemington Road, Parkville, VIC 3010, Australia
6 Faculty of Medicine, Dentistry and Health Sciences, University of Melbourne, Flemington Road, Parkville, VIC 3010, Australia
7 The Open Medicine Foundation ME/CFS Collaborative Research Centre, Bio21 Molecular Science and Biochemistry Institute, University of Melbourne, Melbourne, VIC 3010, Australia
8 Biochemistry Section, Chemistry Department, Faculty of Science, Tanta University, Tanta, Gharbia Governorate, 31527, Egypt
9 Faculty of Medicine, Université Laval, Quebec, QC G1V 0A6, Canada
10 Department of Stomatology, Faculty of Dentistry, Université de Montréal, Montreal, QC H3T 1J4, Canada
* Correspondence: christopher.armstrong@unimelb.edu.au (C.W.A.); alain.moreau.hsj@ssss.gouv.qc.ca (A.M.)

Supplementary Table S1. Comorbidities in Myalgic Encephalomyelitis (ME) patients and healthy controls

**Comorbidities**	**ME cohort N=56 N (%)**	**HC N=16 N (%)**
**Depression**	24 (42.9)	0
**Anxiety**	18 (32.1)	0
**Allergy**	22 (39.3)	4 (7.1)
**Fibromyalgia**	17 (30.4)	0

The number (n) and percentage (%) of participants reporting various comorbidities within the Myalgic Encephalomyelitis (ME) cohort (n=56) and the Canadian healthy control (HC) group (n=16). The comorbidities listed include depression, anxiety, allergy and fibromyalgia.

Supplementary Table S2: Demographic and Clinical Characteristics of Study Participants, Stratified by Sex and Health Status

Characteristics	ME_F (n=37)	ME_M (n=19)	HC_F (n=8)	HC_M (n=8)
Age, years (mean ± SEM)	51 ± 1.6	49 ± 3.7	51 ± 4.4	47 ± 3.9
BMI, kg/m^2 (mean ± SEM)	25.0 ± 0.9	26.0 ± 1.8	23.0± 0.7	27 ± 1.8
Illness duration, years (Mean ± SEM)	13 ± 2.2	14 ± 3.4	N/A	N/A

Demographic and clinical data for patients with Myalgic Encephalomyelitis (ME) and healthy controls (HC), stratified by sex. Data are presented as mean ± standard error of the mean (SEM). The table includes the number of participants for each group (n), as well as measures for age, body mass index (BMI), and illness duration. No statistically significant differences were found between male and female healthy controls for age or BMI, supporting that the sex-specific findings in the ME group are a pathological feature of the disease. Illness duration was not applicable (N/A) to the healthy control groups.

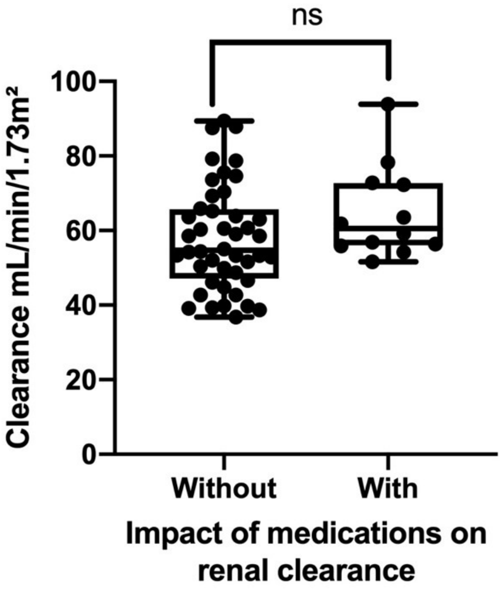

Supplementary Figure S1. Renal Clearance Remains Unaffected by Nonsteroidal Anti-inflammatory Drug (NSAID) Administration. This figure compares renal clearance in two groups of patients with Myalgic Encephalomyelitis (ME): those taking NSAIDs (n = 12) and those not taking NSAIDs (n = 44). No significant difference was observed between these groups (p=0.112) (Mann-Whitney U test).
